# Potential Complementary Effect of Zinc and *Alkalihalobacillus clausii* on Gut Health and Immunity: A Narrative Review

**DOI:** 10.3390/nu16060887

**Published:** 2024-03-19

**Authors:** Rosa María Wong-Chew, Thi Viet Ha Nguyen, Jossie M. Rogacion, Maxime Herve, Etienne Pouteau

**Affiliations:** 1 Infectious Diseases Research Laboratory, Research Division, Facultad de Medicina, Universidad Nacional Autónoma de México, Mexico City 06726, Mexico; rmwong@unam.mx; 2 Department of Paediatrics, Hanoi Medical University, 1,Ton That Tung, Hanoi 116001, Vietnam; vietha@hmu.edu.vn; 3 Department of Gastroenterology, National Children’s Hospital, 18 Lane 879 La Thanh Street, Lang Thuong, Dong Da, Hanoi 116001, Vietnam; 4 Department of Paediatrics, University of the Philippines, Philippine General Hospital, Manila 1000, Philippines; rogacionjossie09@gmail.com; 5 Sanofi-Aventis, 38 Beach Road, Singapore 189767, Singapore; maximehervebkk@gmail.com; 6 Sanofi, 157 Avenue Charles de Gaulle, 92200 Neuilly-Sur-Seine, France

**Keywords:** *Alkalihalobacillus clausii*, dysbiosis, gut microbiota, inadequate zinc intake, probiotics, paediatric zinc deficiency, zinc

## Abstract

A balanced microbiota—microorganisms that live in the gut—is crucial in the early years of a child’s life, while dysbiosis—altered microbiota—has been linked to the development of various diseases. Probiotics, such as *Alkalihalobacillus clausii*, are commonly used to restore the balance of gut microbiota and have shown additional antimicrobial and immunomodulatory properties. Intake of micronutrients can affect the structure and function of the gut barrier and of the microbiota by having multiple effects on cellular metabolism (e.g., immunomodulation, gene expression, and support structure proteins). An inadequate zinc intake increases the risk of deficiency and associated immune dysfunctions; it is responsible for an increased risk of developing gastrointestinal diseases, respiratory infections, and stunting. Paediatric zinc deficiency is a public health concern in many countries, especially in low-income areas. Currently, zinc supplementation is used to treat childhood diarrhoea. This review examines how combining *A. clausii* and zinc could improve dysbiosis, gut health, and immunity. It suggests that this combination could be used to prevent and treat infectious diseases and diarrhoea in children up to adolescence.

## 1. Introduction

The gut contains 70–80% of immune cells, indicating a close relationship between the gut and immune system [1,2]. Microorganisms inhabiting the gut, known as gut microbiota, play a key role in shaping the host immune system, and vice versa. In healthy individuals, the concentration of bacteria in the lower intestine is approximately 10^12^/cm^3^ and is influenced by several factors, including intestinal motility and integrity, gastric acid and pancreaticobiliary secretions, and immunity [3]. The immune system modulates the microbiota, favouring specific bacteria for their important metabolic functions while preventing the proliferation and translocation of harmful species into host tissues and the systemic circulation [4]. Similarly, the microbiota influences host immunity composition, functionality, and effectiveness, including the production of cytokines, antibodies [5], and T cells [6].

The composition of microbiota is affected by various prenatal and postnatal factors (e.g., mode of infant feeding and antibiotic usage), and its maturation starts in early childhood. Balanced microbiota during the first few years are crucial for children’s health [7]. Several studies have demonstrated a correlation between the development of various diseases and an altered microbiota in the gut, a status known as dysbiosis, which involves an imbalance in microorganism composition and functions, as well as changes in metabolic activities, in the large intestine [8]. There is a growing trend towards restoring the gut microbiota balance and symbiotic relationship with the host (eubiosis) using probiotics [9]. Common probiotics belong to different genera, such as lactobacilli, *Bifidobacterium*, *Bacillus*, *Pediococcus*, and several yeasts, and they can be found mainly in dairy foods (e.g., yoghurt) [10]. Some probiotics are used in infant formula to prevent infant colic and promote early cow milk protein tolerance [11]. Strains of *Alkalihalobacillus clausii* (*A. clausii*), previously known as *Bacillus clausii*, are probiotics with antimicrobial and immunomodulatory properties [12,13]. They are useful both for the treatment and prevention of gastrointestinal [14,15,16] and non-gastrointestinal infections [17,18].

The gut microbiota impacts the body’s bioavailability of micronutrients needed for growth and other essential biological activities. At the same time, the intake of micronutrients (e.g., zinc) affects the structure and function of the gut microbiota [19,20]. Zinc is an essential micronutrient, which can be found in a variety of food (meat, fish, eggs, and dairy products) [21], and it can impact gut bacterial survival by modulating the expression of proteins that are necessary for cell survival, such as superoxide dismutase, proteases, DNA polymerases, and ribosomal proteins [22]. It influences bacterial defence and gene expression by inhibiting or promoting factors that impact growth and virulence [23].

In the human body, zinc plays a key role in cellular metabolism as it is the cofactor of numerous enzymes (e.g., hydrolases, transferases, oxidoreductases, ligases, isomerases, and polymerases) [24]. It is also present in the body’s cell surface proteins, preserving the stability and integrity of biological membranes and ion channels, while also contributing to protein synthesis and gene expression [25]. Its multiple properties significantly influence the immune system [26] and the intestinal mucosa [20,27,28,29]. An inadequate zinc intake (which may arise from poor diet, increased organic demand or loss, and decreased absorption) increases the risk of deficiencies and associated immune dysfunctions. Based on dietary intake surveys, zinc deficiency is estimated to be prevalent in most young and adult populations in developed and developing countries, especially in Asia [30]. Paediatric zinc deficiency is prevalent in low- and middle-income countries and is considered a significant risk factor for morbidity, mortality, and stunting [31,32,33]. Preventive zinc supplementation in healthy children could reduce mortality due to common causes like diarrhoea, pneumonia, and malaria [33,34].

Considering the numerous health benefits deriving from zinc (e.g., immunomodulation, preservation of the integrity of biological membranes, and support of protein structure) and *A. clausii* (e.g., antimicrobial and immunomodulation activity), the simultaneous intake of both may help strengthen the developing immune system of children [35,36,37]. This review explores the potential complementary effects of *A. clausii* and zinc with regard to improving dysbiosis, gut health, and immunity, with a possible application in preventing infectious diseases and diarrhoea in children up to adolescence (around 12 years old).

## 2. Gut Microbiota and Dysbiosis

**Figure 1:**
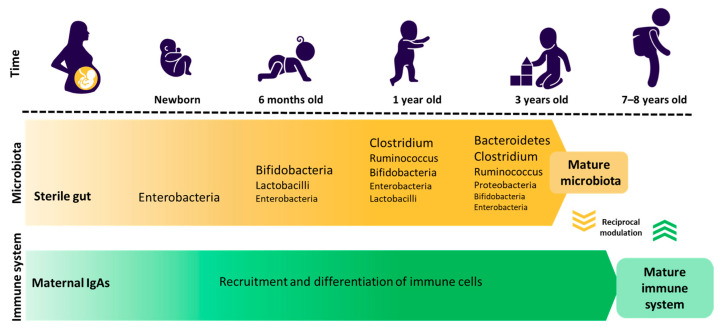
Gut microbiota and immunity development. IgA, immunoglobulin A.

After birth, a newborn’s sterile intestine is colonised by various bacteria (enterobacteria, enterococci, lactobacilli, and bifidobacteria), whose composition undergoes gradual changes until three years of age when the microbiota starts to resemble that of adults, mainly consisting of Firmicutes (50–70%), Bacteroidetes (10–30%), Proteobacteria (up to 10%), and Actinobacteria (up to 10%) [38,39]. At the same age, the young immune system starts producing antibodies, and children begin experiencing significant developmental changes that can impact their health (Figure 1) [39]. For example, once children begin childcare, they are exposed to a variety of bacteria and viruses from the environment and other people.

Mode of birth delivery [40], xenobiotics, and diet can influence the microbial structure [8], disrupting its proper function on intestinal epithelial barrier integrity and enteric nervous system development [41]. In addition, chronic diseases, intense physical and mental stress, prolonged antibiotic therapies, and infections can cause dysbiosis, and harmful microorganisms may take over the gut environment, leading to further multiple diseases (e.g., diarrhoea) [1,7,8,39,42].

### 2.1. Dysbiosis-Related Diseases and Conditions

Numerous studies in the paediatric population have demonstrated the correlation between dysbiosis and various diseases, including chronic inflammatory diseases, metabolic disorders, asthma, infections, cardiovascular issues, neurodegenerative diseases, and autoimmune diseases [39,41,42].

Preclinical studies have shown that the intestinal microbiota composition plays a significant role in weight gain in obesity. The relative abundance of Bacteroidetes and Firmicutes in the gut is associated with the host’s ability to extract energy from their diet. In addition, it has been observed that introducing an ‘obese microbiota’ to germ-free mice caused a significantly greater increase in total body fat compared to those who were introduced to a ‘lean microbiota’ [43].

These findings indicate that the gut microbiota may play a role in obesity development [43]. The development of atopic diseases (e.g., asthma, dermatitis, rhinitis, and food allergy) in children is characterised by a complex interplay of environmental and genetic factors; however, current research highlights the crucial role of gut microbiota [39]. Early studies demonstrated that microbiota affects the development of T helper 1 cells, modulating the balance of T helper 1/T helper 2 cells that is often disrupted in children with atopy during early life [44]. Animal models have assessed the involvement of the gut microbiota in the development of inflammatory bowel diseases, namely Crohn’s disease and ulcerative colitis [45]. Patients with inflammatory bowel diseases consistently exhibit lower bacterial diversity in faecal microbiota samples compared to controls [46]. A case-control study of children aged 2–18 years, and hospitalised with acute ulcerative colitis, showed that the number of microbial phylospecies was reduced in children with ulcerative colitis (266 ± 69) vs. controls (758 ± 3) (*p* < 0.001); likewise, the Shannon Diversity Index (a measure of the diversity of microbiota species) was 6.1 ± 0.23 vs. 6.49 ± 0.04 (*p* < 0.0001), respectively [47].

Changes in the mucosal barrier and tight junctions in the gut during dysbiosis may cause a shift in permeability to microbial products, altering the microbiota, causing leaky gut syndrome, and leading to gastrointestinal diseases, such as diarrhoea, inflammatory bowel disease, irritable bowel syndrome, celiac disease, and necrotising enterocolitis (NEC) [39,48]. NEC occurs mainly in preterm infants whose intestinal barrier is more permeable than full-term newborns. An altered microbiota contributes to the bacteraemia associated with NEC as increased intestinal permeability promotes bacterial translocation into the main circulation. Pathogenic bacteria (e.g., *Enterobacter*) were isolated more frequently from infants diagnosed with NEC compared to controls [49].

Various therapeutic strategies have been used to restore eubiosis, including the use of probiotics, prebiotics, and synbiotics. Administration of probiotics (e.g., *A. clausii*) can prevent the onset of dysbiosis when the patient is exposed to predisposing conditions (medications such as antibiotics or proton pump inhibitors, stress, and diseases) and can act as therapeutic agents to rebalance an existing dysbiosis [9].

### 2.2. Alkalihalobacillus clausii

*A. clausii* is an aerobic, spore-forming bacterium with antibiotic resistance [50]. Strains used in probiotics (O/C, N/R, SIN, and T strains) have complete resistance to some macrolides (erythromycin, azithromycin, clarithromycin, spiramycin), lincosamides (clindamycin, lincomycin), and metronidazole [50]. However, each strain shows different degrees of resistance to other antibiotics; O/C is resistant to chloramphenicol, N/R is resistant to novobiocin and rifampicin, T is resistant to tetracycline, and SIN is resistant to neomycin and streptomycin [50]. The potential risk of subsequent transfer of antibiotic resistance from *A. clausii* to pathogenic microorganisms has been deemed very low based on numerous studies [17,51]. Therefore, these strains have been routinely used for over 60 years for restoring the gut microbiota during antibiotic treatments. *A. clausii* can survive in the gastrointestinal tract at different pH conditions and proliferate up to 12 days after oral supplementation [52].

Although the molecular basis of the properties of *A. clausii* is still unclear, several studies have identified potential mechanisms of action for the variety of its effects (antimicrobial and immunomodulatory effects, cell growth and differentiation regulation, cell–cell communication, cellular adhesion, and gut homeostasis maintenance) [53,54].

*A. clausii* may alter the microbiota composition by direct contact or by producing bacteriocins, which are small, potent antimicrobial peptides [12]. Antimicrobial substances were observed in *A. clausii* media, which displayed Gram-positive antibacterial action [55]. In addition, antimicrobial peptides were secreted by *A. clausii* cells cultured in whey, and they inhibited the growth of *Salmonella typhimurium*, *Escherichia coli*, *Shigella flexneri*, *Staphylococcus aureus*, *Listeria monocytogenes*, and *Enterococcus faecalis*, indicating preventive action against intestinal infections [53]. The O/C strain of *A. clausii* has demonstrated the ability to produce lantibiotics, which are post-translationally modified antimicrobial peptides able to prevent the growth of pathogenic bacteria in the gastrointestinal tract. Specifically, the lantibiotic clausin exhibited antimicrobial activity against some Gram-positive bacteria and inhibition of the toxic effects of *Clostridioides difficile* [56].

The immunomodulatory activity of *A. clausii* has been demonstrated in clinical settings. In allergic children with recurrent respiratory infections, the beneficial effects were shown by an increase in cytokines (interferon gamma [IFN-γ] and interleukin-(IL)12, which downregulated allergic reactions, and by a decrease in cytokines (IL-4), which promoted inflammation during allergy [35]. A preclinical study using an enterocyte model of rotavirus infection demonstrated the efficacy of *A. clausii* in paediatric viral acute gastroenteritis via multiple mechanisms of action [57]. The probiotic induced the synthesis of human β-defensin 2 and cathelicidin (antimicrobial peptides), stimulated cell proliferation, and reduced the proportion of necrotic or apoptotic enterocytes.

*A. clausii* is also involved in gut homeostasis, improving gut barrier integrity by increasing mucin production and the synthesis of tight junction proteins [58]. The protective activity of *A. clausii* against the cytotoxic effects of *Clostridium difficile* and *Bacillus cereus* strains, microbes involved in the onset of diarrhoea in children, has been demonstrated. The in vitro coincubation of toxic culture of pathogens and *A. clausii* strain O/C completely prevented the damage induced by toxins in Vero and Caco-2 cells due to the alkaline serine M-protease secreted by *A. clausii* [56].

Various studies have assessed the safety of *A. clausii* in children. A prospective, single-blind, randomised, controlled trial conducted in 2007 to evaluate the effects of *A. clausii* in the management of diarrhoea in children (N = 192) aged 3–36 months demonstrated that the administration of 2 × 10^9^ spores of *A. clausii* for 5 days was well-tolerated with no adverse events [59]. In the same year, the safety of *A. clausii* (2 billion spores/5 mL for 3 months) was confirmed in older children aged 3–6 years with recurrent respiratory infections [18]. In a population-based study in Filipino children with acute diarrhoea (N = 3178 patients, median age of 2 years), the safety and effectiveness of *A. clausii*, as an adjunct to standard therapy, were assessed. Treatment with *A. clausii* was well-tolerated, with 3/3178 (0.09%) mild to moderate adverse events reported, including vomiting, rash, and stool colour change [14]. *A. clausii* is considered well-tolerated for children at a daily dose of 2 × 10^9^ colony forming units [CFUs] per day, and up to 3 months of supplementation [18,59]. Side effects (e.g., systemic infections) have been inconsistently reported in very few cases and not adequately assessed [60,61,62]. However, surveillance for adverse events should always be considered, particularly in vulnerable populations (e.g. intensive-care settings).

## 3. Zinc

### 3.1. Dietary Needs and Biological Role

Zinc is an essential mineral with a critical role in childhood growth [63], and the correct intake should be ensured for healthy individuals. Expert committees of the World Health Organization (WHO) [64], Institute of Medicine (IOM) [65], International Zinc Nutrition Consultative Group (IZiNCG) [66], and European Food Safety Authority (EFSA) [29] proposed values for zinc-recommended daily allowances (Table 1) [67].

Zinc is involved in multiple physiological processes, including immunity, inflammation, and epithelial integrity [68]. It is critical for the maturation of the immune system in children after 3–4 years of age [26,27,29] and reaches its full potential to support immunity in children of 7–8 years of age when the immune system is mature [28,37]. Many proteins involved in antiviral defence contain zinc in their structure as a cofactor; consequently, zinc has a significant impact on the antiviral immune response and immune regulation, in particular, in the gastrointestinal and respiratory tract [69,70]. It plays an essential role in the signal transduction pathways that eliminate pathogens by promoting neutrophil extracellular trap formation and inducing cell-mediated immunity over humoral immunity [71]. Zinc influences the inflammation process, modulating the proinflammatory response by targeting inflammatory cytokines and nuclear factor kappa B (NFκB), a crucial mediator in inflammation, which controls apoptosis, cell adhesion, proliferation, tissue remodelling, innate and adaptive immune responses, and cellular stress responses. Zinc also protects cells from oxidative damage through various mechanisms of action, including the stabilisation of membranes, inhibition of pro-oxidant enzymes (nicotinamide adenine dinucleotide phosphate oxidase), and synthesis of antioxidant enzymes (metallothioneins) [72].

Zinc plays an important role in intercellular junction proteins, which are the structures that promote adhesion between epithelial cells and are necessary for epithelial tissue structure and selective barrier function [73]. Recent evidence has shown that zinc transporters contribute to the barrier function of intestinal epithelial cells and respiratory epithelium [69,74]. These epithelial barriers need to maintain their integrity to avoid exposing aseptic internal organs and the bloodstream to external harmful agents [75].

### 3.2. Prevalence and Health Consequences of Inadequate Zinc Intake and Zinc Deficiency

#### 3.2.1. Prevalence of Inadequate Zinc Intake

Adequate zinc intake is necessary for normal child growth, immune function, and neurological development. Inadequate intake of zinc can occur due to various reasons, such as decreased intake, an inability to absorb micronutrients, excessive loss, or increased demand [31]. Decreased intake may be due to poor diet (lack of food availability in low-income countries), lack of meat intake, excess phytates (legumes, seeds, and soy products), or oxalates (spinach, nuts, and tea). Phytates and oxalates form insoluble complexes with zinc, reducing their bioavailability in the digestive tract [76]. Gastrointestinal diseases (e.g., Crohn’s disease, short bowel syndrome, and pancreatic insufficiency) and some medications (e.g., penicillamine, diuretics, antibiotics, and sodium valproate) can cause inadequate absorption, while burns, haemolysis, diarrhoea, or urinary excretion due to diuretic use can cause excess loss. Preterm infants require increased zinc due to inadequate stores, decreased gut absorption, and higher metabolic rates [77].

The evaluation of zinc status in a particular population is commonly determined by examining circulating zinc levels [78]. However, as a result of a lack of standardisation in sampling procedures, there have been inconsistencies in the results obtained, and different methods have been developed to assess the prevalence of inadequate global zinc intake [30]. In 2001, the estimated prevalence of low zinc intake was 49% [79], while an analysis in 2005 estimated the prevalence to be 21% [80]. The latest estimation carried out using the dietary requirement recommendation from the Food and Nutrition Boards of the IOM and the IZiNCG established that up to 17% of the global population is at risk of inadequate zinc intake [30]. When inadequate zinc intake prevalence is greater than 25% in a population, the risk of zinc deficiency is considered elevated and a public health concern [30,81]. Regions at major risk of zinc deficiency include South Asia, sub-Saharan Africa, and Central America (Table 2) [30].

#### 3.2.2. Prevalence of Zinc Deficiency

Zinc deficiency may arise from a combination of factors, including inadequate intake, mostly caused by poor diet, diminished absorption, or increased intestinal loss resulting from inflammatory processes (e.g., bowel diseases) [82]. Around 50% of children in low- and middle-income countries may have zinc deficiency due to limited food availability [33]. Paediatric zinc deficiency is a public health concern in many countries [66]. Zinc deficiency accounts for the majority of direct medical costs for the treatment of diarrhoea, respiratory disease, and measles [83,84,85]. In 2017, zinc deficiency affected 83% of children in Cameroon, 25–30% in the Philippines [84], 58% in Vietnam [86], and 28% in Turkey [63]. Similar results were observed in a study conducted in 2014 by the National Institute of Nutrition in Vietnam among 466 children (6–59 months of age) with anorexia, of whom 47% also had anaemia and 44% zinc deficiency, while 21% had both anaemia and zinc deficiency [87]. A recent study mapped micronutrient deficiencies following the Sustainable Micronutrient Interventions to Control Deficiencies and Improve Nutritional Status and General Health in Asia program in five South-East Asian countries (Cambodia, Indonesia, Laos PDR, Vietnam, and Thailand) and found that the coverage of zinc supplementation was too low to contribute to the prevention of deficiency [88]. Limited studies are available on the prevalence rates of zinc deficiency in healthy young children from Western high-income countries [89,90,91,92]. In 2021, a study demonstrated that zinc deficiency is common (32%) in healthy 1- to 3-year-old children in Western European countries (Germany, The Netherlands, and the UK) [91].

#### 3.2.3. Health Consequences of Inadequate Zinc Intake and Zinc Deficiency

Inadequate zinc intake may present a variety of clinical manifestations (growth retardation, rough skin, poor appetite, mental lethargy, abnormal neurosensory changes, and delayed wound healing) depending on the severity of the condition, and it may lead to zinc deficiency [93]. Zinc deficiency in children is responsible for an increase in the risk of developing gastrointestinal diseases (especially diarrhoea) [20,37,82,94,95,96], acute lower respiratory tract infections [27,69,70,97,98,99], and growth retardation [28,88,100]. The influence of zinc deficiency on gastrointestinal health derives from a variety of mechanisms, including the direct alteration of gut microbiota, decreased immunity, and impaired barrier integrity [37,81,101]. In a preclinical study (*Gallus gallus* model), zinc deficiency induced significant taxonomic alterations in the gut, creating a microbial profile similar to pathological states. In particular, it increased the abundance *of Enterococcus* and *Ruminococcaceae* and decreased that of *Clostridiales* and *Peptostreptococcaceae*, while decreasing the richness and diversity of the overall species [96]. The expert panel of the EFSA has established a cause–effect relationship between zinc dietary intake and normal immune function [29]. Zinc deficiency promotes thymic atrophy and lymphopenia, and it decreases innate and adaptive immunity. It impairs the host defence by compromising phagocytosis, intracellular killing activity, and cytokine production by macrophages [68]. In addition, an increase in inflammatory response and damage to host tissue with the production of IL-1, IL-6, and tumour necrosis factor-α (TNF-α) is observed when zinc concentration is low [71]. Likewise, a lack of zinc alters the antibody secretion of T and B cells [95,102]. A study on school-age children with zinc deficiency showed a significant increase in inflammatory cytokines in the zinc deficiency group compared to the control group (*p* < 0.05) [20]. Under zinc deficiency conditions, the disruption of intercellular junctions occurs, with a consequent reduction in tissue integrity and impairment of the control of paracellular permeability, leading to leaky gut syndrome and the passage of pathogens into the bloodstream [73].

Mucosal barrier dysfunctions are not limited to the gut; they also occur in the respiratory tract. Zinc depletion in airway epithelial cells disrupts the structural proteins, including β-catenin and E-cadherin, leading to enhanced leakage across the respiratory epithelial barrier [69]. A study conducted in 2018 revealed that serum zinc levels in paediatric patients with pneumonia were significantly lower among those admitted to the intensive care unit than those admitted to other wards (*p* < 0.001). The study also found a statistically significant decrease in zinc levels in critically ill children with sepsis, mechanically ventilated cases, and fatal cases (all *p* < 0.001) [99].

Short stature and poor weight gain have been reported in children with zinc deficiency [99,103,104]. The mechanisms behind growth retardation caused by a lack of zinc are not yet fully understood. One hypothesis suggests that zinc deficiency may reduce the synthesis and secretion of growth hormone (GH) and/or insulin-like growth factor-1 (IGF-1) [69,105]. An in vitro analysis of GH secretion showed that a reduction in intracellular-free zinc content may interfere with normal GH dimerisation, resulting in impaired secretion [105]. A preclinical study demonstrated the association of serum zinc concentration with inadequate growth and reduced IGF-1. In rats on zinc-deficient fodder for 14 days, weight gain was reduced by 83% compared to pair-fed controls. Serum zinc, IGF-1, and insulin were reduced by 80%, 69%, and 66%, respectively [106].

Scientific evidence has shown a relationship between zinc and anaemia [107]. Although animal models have suggested that zinc plays a significant role in erythropoiesis [108], a direct correlation between zinc concentration and the production of red blood cells in humans has not been established. Nevertheless, zinc is directly involved in erythroid differentiation and development [109]. A cross-sectional study involving 243 children aged 12–72 months in rural Vietnam found that 87% of children were deficient in zinc, and a combination of anaemia and zinc deficiency occurred in 45% [110]. A multi-country study (>13 low- and middle-income countries) demonstrated that zinc concentrations were independently and positively associated with haemoglobin concentrations in preschool children and nonpregnant women of reproductive age [101].

### 3.3. Zinc Supplementation

Considering the plethora of biological processes involving zinc, it is not surprising that zinc supplementation has been assessed in studies to treat or prevent various diseases.

#### 3.3.1. Zinc Supplementation for Disease Treatment

The benefits of zinc in treating gastrointestinal diseases have been broadly recognised for over 20 years [111]. The efficacy of zinc supplementation as a treatment of childhood diarrhoea has been evaluated mainly in low-income countries, while the effect on diarrhoea in well-nourished children needs to be clarified. A meta-analysis, including 33 trials mainly conducted in Asia (N = 10,841; children aged 1 month to 5 years), showed that in children >6 months, zinc supplementation shortened the mean duration of diarrhoea (−11.46 h, 95% confidence interval [CI] −19.72, −3.19) and reduced the number of children who experienced prolonged diarrhoea (RR 0.73, 95% CI 0.61, 0.88) [112]. The WHO and the United Nations Children’s Fund recommend short-term zinc supplementation of 20 mg zinc per day or 10 mg for infants under 6 months for 10–14 days to treat acute childhood diarrhoea [113].

Treatment with zinc supplementation has also shown efficacy against viral (herpes simplex, common cold, and viral warts) and bacterial infections in children [98,114]. A randomised controlled clinical trial on children aged 1 month to 5 years old, and hospitalised with pneumonia, showed that zinc supplementation improved their clinical status (mean in hours (h) of the combination of all the clinical variables ± standard error [SE]) (76 ± 7 vs. 105 ± 8, *p* = 0.01), respiratory rate (37 ± 6 vs. 57 ± 7, *p* = 0.04) (h ± SE), and oxygen saturation (53 ± 7 vs. 87 ± 9, *p* = 0.007) (h ± SE) in the intervention group compared to the placebo group, and promoted a Th1 immune response [114].

A retrospective study on children (N = 114; aged 4 months to 6 years) diagnosed with failure to thrive (inadequate ponderal or linear growth) showed that zinc supplementation (10 mg/day for 6 months) increased growth and serum zinc levels according to undernutrition severity. The supplementation was more effective in underweight (weight below the recommended level for a specific age) children than stunting (height below the recommended level for a specific age) children, suggesting an important role in catch-up growth by significantly increasing serum zinc concentrations at the early stages of growth retardation [115]. Daily supplementation of 5 mg elemental zinc for 6 months has shown to be effective in improving physical growth (height increment and weight gain) in children (aged 2–5 years) with retarded linear growth, especially in boys (*p* = 0.001). The stunted growth rate changed significantly (*p* = 0.01) from 27% to 3% throughout the study [116].

Zinc supplementation has been proposed for managing patients affected by coronavirus disease 2019, as it may help overcome the severe acute respiratory syndrome coronavirus 2 (SARS-CoV-2) infection by boosting the immune response and inhibiting viral replication and genome transcription [97]. Zinc treatment may act synergistically with standard antiviral therapy in patients with hepatitis C, human immunodeficiency virus, and SARS-CoV [69,117]. Data from the Medical Informative Mart for Intensive Care-IV database have been used for a meta-analysis that showed the association of zinc supplementation with reduced in-hospital mortality (hazard ratio = 0.48, 95% CI 0.28, 0.83; *p* = 0.009) in critically ill patients with acute kidney injuries [118].

#### 3.3.2. Zinc Supplementation for Disease Prevention

Although the main use of zinc supplementation is for treating the malnourished paediatric population, the number of studies reporting positive outcomes as a preventive intervention in healthy children is growing [33,34,104,116,119,120,121,122].

In 2009, preventive zinc supplementation was evaluated among infants, preschoolers, and older prepubertal children. Results from the meta-analysis of 55 trials performed in different countries (seven in Africa, 23 in Asia, 12 in South America, 11 in North America, one in Australia, and one in Europe) on 202,692 children showed that, in children receiving zinc supplementation compared to control groups, the incidence of diarrhoea was reduced by 20%; acute lower respiratory tract infections by 15%; and deaths by 18% in children aged ≥1 year (but it had no effect in younger children). In addition, a small increase in linear growth and weight was observed in all children [120].

Zinc supplementation has been evaluated as a preventive intervention to reduce mortality due to common causes, such as diarrhoea, pneumonia, and malaria [33,34,121]. A meta-analysis of 96 studies on children aged 6 months to 11 years (N = 219,584) showed no difference in all-cause mortality with preventive zinc supplementation compared to no zinc (risk ratio [RR] 0.93, 95% CI 0.84, 1.03). However, evidence of moderate certainty demonstrated that zinc supplementation may reduce mortality due to lower respiratory tract infections (RR 0.86, 95% CI 0.64, 1.15) and malaria (RR 0.90, 95% CI 0.77, 1.06) [33]. The same study assessed that preventive zinc supplementation would likely reduce the incidence of all-cause diarrhoea (RR 0.91, 95% CI 0.90, 0.93) and lead to a slight increase in height (standardised mean difference 0.12, 95% CI 0.09, 0.14) [33].

A randomised, double-blind trial on healthy children aged 6–24 months assessed the association of preventive zinc supplementation for 6 months with growth, and it showed a significant difference in the average length increment in the intervention group compared with the placebo group (placebo 5.23 ± 2.19 vs. zinc 5.79 ± 2.18 cm, *p* = 0.02). The beneficial outcomes on growth and average length increment suggest a strategy for preventing growth retardation and stunting [119].

Zinc supplementation in children and adolescents has not been associated with serious safety concerns. However, excessive intake may cause minor side effects, such as vomiting [123,124]. Therefore, tolerable upper intake levels for children have been established (Table 3) [125,126].

## 4. Benefits of the Combination of Zinc and *A. clausii*

Considering the wealth of published data on the distinct actions of zinc and probiotics in preventing and treating gastrointestinal and respiratory diseases, it is important to investigate the effects of co-administration.

A number of studies on the combination of different probiotics and micronutrients have been conducted and have resulted in promising outcomes [15,36,127,128,129,130]. A positive effect on mild diarrhoea has been demonstrated with the supplementation of probiotics and zinc in children aged ≤1 year [130]. A double-blind, randomised trial showed a significant reduction in the duration of diarrhoea in 65 infants (aged 6–12 months) treated with a combination of probiotics (*Streptococcus thermophilus*, *Bifidobacterium lactis*, *Lactobacillus acidophilus*, 6 × 10^9^ colony-forming units), zinc (10 mg/day), and fructo-oligosaccharides (0.3 g). Children in the intervention group had diarrhoea resolution after 1.43 ± 0.71 days vs. 1.96 ± 1.24 days in children in the control group (*p* = 0.017). After 3 days, the number of children with watery stools was 1 vs. 10 in the supplemented and control groups, respectively (*p* = 0.02). A subset of children also suffering from vomiting had a time to resolution of vomiting of 0.27 ± 0.59 days vs. 0.81 ± 0.91 days in the supplemented and control groups, respectively (*p* = 0.06). A combination of probiotics with zinc and selenium for the treatment of paediatric rotavirus enteritis was assessed in a retrospective study that enrolled 85 children from January 2019 to December 2020. The children received probiotic therapy (*Bifidobacterium*) three times a day. After the intervention, a significant clinical benefit (*p* < 0.05) was observed: the total effective rate of the two groups was 88.4% (38/43) and 50% (21/42), respectively, and the time to disappearance of symptoms (fever, vomiting, diarrhoea, and dehydration) was shorter in the experimental group compared to the control group. Similarly, levels of inflammatory factors (IL-6, IL-8, and high-sensitivity C-reactive protein) in the experimental group were significantly lower than those in the control group (all *p* < 0.05) [127].

In 2015, a prospective study on *A. clausii* as an add-on therapy to oral rehydration therapy (ORT) and zinc supplementation in the management of acute diarrhoea was performed [128]. Children (N = 131) admitted to a paediatric ward in Navi Mumbai, India, with acute diarrhoea were included in the analysis and divided into two groups. Group 1 (N = 69) included children on combination therapy of *A. clausii* (oral suspension of 2 billion spores per 5 mL bottle) ORT, and zinc; Group 2 included children only on ORT and zinc. Group 1 experienced a more rapid decrease in the frequency of diarrhoea (24 h vs. 60 h, *p* < 0.01) and shorter hospital stay (2.78 days vs. 4.30 days) compared with Group 2 [128]. In 2022, a randomised, double-blind trial assessed the efficacy and safety of *A. clausii* combined with oral ORT and zinc supplementation in acute diarrhoea in 454 children (aged 6 months to 5 years). Participants in the treatment arm received *A. clausii* (oral suspension of 2 billion spores per 5 mL bottle) for 5 days plus ORT and zinc, while children in the control group received only ORT and zinc. Similar proportions of patients showed recovery from diarrhoea over 120 h after randomisation, and the combination was well-tolerated, with an incidence of adverse events of 9.7%, similar to that of children in the placebo group (12.3%) [15]. While the results from the 2015 study showed a promising role of *A. clausii* as an add-on therapy to ORT and zinc in the management of acute diarrhoea in children, the beneficial effects were unclear in the subsequent clinical trial. Both studies enrolled children with mild to moderate dehydration and administered the same dose of *A. clausii*. Although the 2015 study had an older population compared to the 2022 study (6 months to 12 years vs. 6 months to 5 years), most children in both studies were under two years old, the age with the highest incidence of rotavirus diarrhoea. In India, rotavirus vaccination was introduced in 2016 as part of the national immunisation program [129]. So, the beneficial effect observed in the 2015 study may have been due to the efficacy of *A. clausii* against rotavirus [57], while the absence of a treatment effect in the 2022 study may be attributed to the impact of rotavirus immunisation. However, further trials are needed to determine the effectiveness of this therapy.

Nevertheless, given the evidence of a well-tolerated *A. clausii*–zinc combination [15], hypotheses suggest that this combination may provide synergistic or complementary benefits for gut barrier integrity [131] and immunity [36], leading to greater benefits compared to using them separately. However, further research is required to understand the potential of their combined effects and mechanisms of action.

### 4.1. Mechanism of Action: Potential Complementarity of Zinc and A. clausii

**Figure 2:**
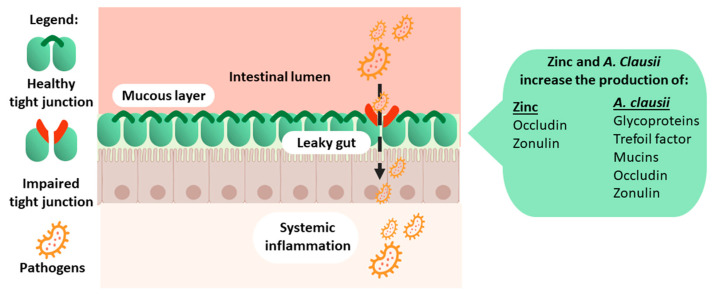
Healthy gut, barrier integrity, and immunity.

Zinc and *A. clausii* play a crucial role in developing a healthy gut in children, thereby helping avoid leaky gut syndrome and opportunistic infections that trigger the immune system (Figure 2). Compromised gut integrity enables the passage of luminal pathogens into the mucosa, which activates the local immune system, while damage-associated molecular patterns are translocated into the liver, triggering systemic inflammation [74].

*A. clausii* favours the expression of adherence molecules and the production of mucins, glycoproteins that are essential to gut barrier integrity. It induces messenger RNA (mRNA) expression of major proteins involved in tight junctions (occludin and zonulin 1) and of a protective factor, trefoil factor 3, which is mainly involved in maintaining and repairing the intestinal mucosa [58]. In addition, *A. clausii* showed the ability to regulate immune and inflammatory functions. In a preclinical study on mice with antibiotic-induced intestinal injury, *A. clausii* induced an increase in mRNA levels of different inflammatory cytokines, such as IL-1β and TNF-α, and a reduction in the anti-inflammatory IL-10. Consistently, the mRNA of chemokine ligand 2 and integrin alpha X (markers of innate immune cell infiltration in the colon) was reduced [58].

**Figure 3:**
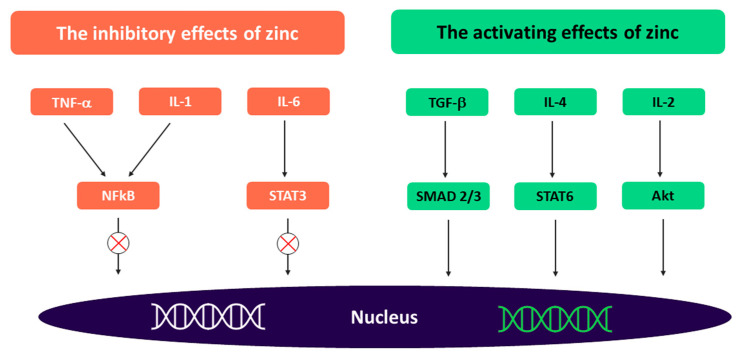
Modulation of the immune function by zinc. Akt, protein kinase B IL, interleukin; NFkB, nuclear factor kappa-light-chain-enhancer of activated B cells; TGF, transforming growth factor; TNF, tumour necrosis factor; SMAD; signalling mothers against decapentaplegic; STAT, signal transducer and activator of transcription. Adapted from Maywald et al. [37].

Zinc is crucial to the resistance of epithelial cells and barrier integrity due to its direct influence on cell tight junctions [94,132] and indirect modulation of microbiota [133]. In particular, zinc reduces pathogenic strains of *Enterobacteria* and *Clostridial cluster* XIV and *E. coli* [134], with a subsequent decrease in the expression of alpha-haemolysin, which is responsible for intestinal barrier dysfunction and, consequently, leaky gut [135]. Zinc promotes the growth of B and T lymphocytes, leading to improved innate and adaptive immunity. Paediatric clinical trials have demonstrated that cell-mediated immunity is improved in young children after 3–4 months of daily zinc supplementation [136,137,138]. A randomised, double-blind placebo-controlled trial on children (1–5 years old) with Shigella infection demonstrated that 2 weeks of zinc supplementation increased lymphocyte proliferation (*p* = 0.002) and antigen-specific antibodies (*p* < 0.001) in the intervention group compared to the control group [137]. An increase in circulating B lymphocytes was observed in a subsequent study on malnourished children with acute shigellosis treated with elemental zinc as adjunct therapy [136]. In vitro studies have demonstrated that zinc balances pro- and anti-inflammatory cytokines [71,72]. A potential mechanism responsible for cytokine regulation has been hypothesised by Maywald et al. [37]. Zinc may have an inhibitory effect on proinflammatory pathways, preventing the nuclear translocation of the nuclear factor kappa-light-chain-enhancer of activated B cells and the subsequent expression of proinflammatory cytokines, while inhibiting the IL-6-mediated activation of the signal transducer and activator of transcription 3, an important mediator in cell proliferation and differentiation in inflammatory diseases. In addition, zinc may activate the immune signalling pathways (transforming growth factor-β, IL2, and IL4) responsible for fighting infections (Figure 3).

The combined immunomodulatory effects of zinc and *A. clausii* can provide additional beneficial outcomes.

### 4.2. Potential Clinical Applications

**Figure 4:**
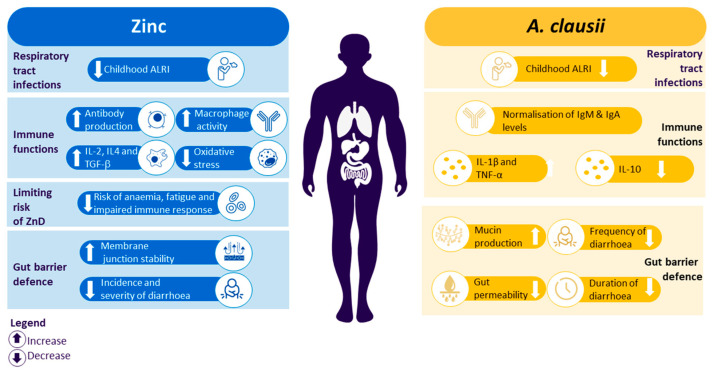
Combination of zinc and *A. clausii* mechanism of action: dual care for gut health and immunity. ALRI, acute lower-respiratory infections; Ig, immunoglobulin; IL, interleukin; TGF, transforming growth factor; TNF, tumour necrosis factor; ZnD, zinc deficiency.

The mutual influence and effects on microbiota homeostasis make zinc and *A. clausii* perfect candidates for a combination formulation to prevent and treat dysbiosis and related diseases, such as diarrhoea and infections (Figure 4). Zinc may help reduce childhood respiratory infections by promoting immune function (by increasing lymphocyte ratios, antibody production, and macrophage activity, which helps fight oxidative stress), and it may help fight gastrointestinal diseases by improving the defence of the gut barrier (by improving membrane junction stability, therefore reducing the incidence and severity of diarrhoea). *A. clausii* may provide beneficial effects in mucosal (respiratory and gastrointestinal) protection (mucin production) and immunity (normalisation of immunoglobulin levels), and it may help reduce the severity of diarrhoea. While probiotics are already extensively used as a preventive intervention against gastrointestinal issues or to boost immunity, zinc supplementation is still mainly used as a treatment in populations with inadequate dietary intake. However, scientific evidence for zinc as a preventive intervention is growing, alongside knowledge of the complementary modes of action of zinc and probiotics, which represents a shift from the traditional use of zinc supplementation as a treatment. This may pave the way for the use of zinc alongside probiotics as a preventive intervention to improve children’s health.

## 5. Conclusions

*A. clausii* has antimicrobial and immunomodulatory properties and plays a crucial role in cell growth and maintaining gut homeostasis. Zinc is an essential micronutrient for human metabolism, and it is involved in numerous biological pathways and immune functions. The combination of zinc and *A. clausii* may contribute to the eubiotic balance of intestinal flora and help prevent cells from incurring abnormal metabolism and damage. In addition, their combined effects on the immune system may provide additional benefits in preventing and fighting intestinal and respiratory infections.

Given the evidence of a possible synergistic or complementary action of zinc and *A. clausii*, together with their good tolerability, which has already been widely demonstrated, this combination is recommended for children. However, further clinical studies are needed to fully demonstrate the efficacy of this combination.

## Figures and Tables

**Table 1:** Zinc-recommended daily allowances in children.

EFSA		IOM		IZiNCG		WHO	
Age, Sex	RDA, mg/day	Age, Sex	RDA, mg/day	Age, Sex	RDA, mg/day	Age, Sex ^a^	RDA, mg/day
	–	0–6 months	2.0 ^b^		–	0–6 months	2.8
7–11 months	2.9	7–12 months	3.0	6–11 months	4.0	7–12 months	4.1
1–3 years	4.3	1–3 years	3.0	1–3 years	3.0	1–3 years	4.1
4–6 years	5.5	4–8 years	5.0	4–8 years	4.0	4–6 years	4.8
7–10 years	7.4					7–9 years	5.6
11–14 years	9.4	9–13 years	8.0	9–13 years	6.0		–
15–17 years, M	12.5	14–18 years, M	11.0	14–18 years, M	10.0	10–18 years, M	8.6
15–17 years, F	10.4	14–18 years, F	9.0	14–18 years, F	9.0	10–18 years, F	7.2

EFSA, European Food Safety Authority; F, female; IOM, Institute of Medicine; IZiNCG, International Zinc Nutrition Consultative Group; M, male; WHO, World Health Organization. ^a^ Data indicated when available; ^b^ Adequate intake (i.e., insufficient evidence for establishing an RDA).

**Table 2:** Areas at high risk of zinc deficiency based on the estimated prevalence of inadequate zinc intake.

Region at High Risk of Zinc Deficiency	Country	Estimated Prevalence of Inadequate Zinc Intake (%)
South Asia	Indonesia	31
	Sri Lanka	31
	India	31
Sub-Saharan Africa	Guinea-Bissau	27
	Kenya	25
	Liberia	35
	Malawi	41
	Zambia	45
	Zimbabwe	48
Central America	El Salvador	28
	Guatemala	30
	Nicaragua	29

Data from country-specific food balance data. Adapted from Wessells KR et al. [30].

**Table 3:** Tolerable upper intake levels of zinc in children.

	EFSA	IOM
Age, Years	ULs, mg/day	
0–6 months	-	4
7–11 months	-	5
1–3 years	7	-
4–6 years	10	-
4–8 years	-	12
7–10 years	10	-
9–13 years	-	23
11–14 years	18	-
14–18 years	-	34
15–17 years	22	-

EFSA, European Food Safety Authority; IOM, United States Institute of Medicine; ULs, upper intake levels. - Not available.

## Data Availability

Not applicable.
